# Complex Network Analysis for Characterizing Global Value Chains in Equipment Manufacturing

**DOI:** 10.1371/journal.pone.0169549

**Published:** 2017-01-12

**Authors:** Hao Xiao, Tianyang Sun, Bo Meng, Lihong Cheng

**Affiliations:** 1 Department of Economics and Trade, Hunan University, Changsha, Hunan Province, China; 2 Department of Institute of Developing Economies, Japan External Trade Organization, Chiba-shi,Chiba, Japan; Tianjin University, CHINA

## Abstract

The rise of global value chains (GVCs) characterized by the so-called “outsourcing”, “fragmentation production”, and “trade in tasks” has been considered one of the most important phenomena for the 21st century trade. GVCs also can play a decisive role in trade policy making. However, due to the increasing complexity and sophistication of international production networks, especially in the equipment manufacturing industry, conventional trade statistics and the corresponding trade indicators may give us a distorted picture of trade. This paper applies various network analysis tools to the new GVC accounting system proposed by Koopman et al. (2014) and Wang et al. (2013) in which gross exports can be decomposed into value-added terms through various routes along GVCs. This helps to divide the equipment manufacturing-related GVCs into some sub-networks with clear visualization. The empirical results of this paper significantly improve our understanding of the topology of equipment manufacturing-related GVCs as well as the interdependency of countries in these GVCs that is generally invisible from the traditional trade statistics.

## Introduction

Complex networks are a modern way to characterize mathematically a series of different systems in the shape of subunits (nodes) connected by their interaction (edges). The complex network approach has gained increased attention from a growing number of researchers interested in examining the structural and dynamical properties involving networks in a wide variety of disciplines, such as nonlinear analysis [1–5], social network analysis in sociology [6], biology [7], economics [8], and many more. Moreover, a key question in network science concerns the topological measures utilized to define the properties of the network connecting the agents, and in what way these properties influence the behaviour of the agents as well as the evolution of the system analysed [9].

As an frontier research into the application area of complex network analysis, international trade networks are a vivid demonstration of economic interactions and linkages among countries and regions. Network analyses have been extensively adopted in international trade study [10–14]. However, as the international division of labour led by multinational corporations has transformed intra-industry trade into intra-product trade [15], the production process of final goods has been split into various tasks and activities across countries. Consequently, intermediate goods cross borders multiple times before they are consumed by final users indicating the increasing number of final goods “Made in the World” [16] and the importance of GVCs.

The concept of GVCs was initially proposed by Krugman (1995) [17], indicating that in international production networks, each country gains value-added amounts by participating in certain production phases. Because of the increasing complexity and sophistication in GVCs, the traditional approaches to explaining global trade face many new challenges as mentioned in Maurer and Degain (2010) [18] that “what you see is not what you get”. There are many responses to these challenges.

Hummels et al. (2001; HIY) [19] defined vertical specialization (VS) and proposed the measurement of “import contents of export” in the context of GVCs. Following that, Daudin (2011) [20] proposed the DRS method that later applied and extended to empirical studies on main OECD countries [21], United States [22], and China [23–25]. In addition, based on the Leontief insights into the input-output linkage between the gross output and the final demand, the value-added export that can be derived by multiplying the gross output caused by a particular final demand with the value-added to gross output ratio in each country/industry is another important index to measure the value-add in domestic and foreign goods [26].

However, the export also includes the intermediate export—the value-added export is part of the export. Wang et al. (WWZ, 2013) [27] decomposed intermediate trade into value-added and double-counted parts and then established a transparent framework to bridge the gap between official trade (in gross output terms) and national accounts statistics (in value added terms). The full decomposition of the export data at bilateral, sector levels can reshape our understanding of the pattern of global trade. In particular, the value-added structure and double counting of gross trade can be used to re-evaluate a country/sector’s position and participation in global production chains. For example, China imports intermediate goods embed USA’s value from the USA and re-exports to USA after processing. This can be explained as foreign value in China's export or the domestic value finally returning USA in its export. The method provides a more objective evaluation of value-added gained by exports and the embedded value-added flows in gross trade, and also better clarifies the fragmentation of global production and the distribution of trade gains. One of its applications is that the OECD-WTO has set up the so-called TiVA (Trade in Value-added) indicator system [28].

In contrast to conventional trade analyses, international trade under the GVC framework involves not only final goods trade, but also the complex production networks of intermediate goods embodied in trade by various routes, etc. To some degree, the way to better understand a country's position in the GVCs is in view of various trade linkages among countries. This leads to many unresolved doubts: “What do GVC networks looks like? How do the GVC networks differ from traditional trade networks? What are the special features and evolutionary trends of GVC networks?”, etc.

To the best of our knowledge, there are only limited studies on global trade networks through a combined approach of GVC accounting and complex network analysis. Ferrarini (2013) calculated bilateral vertical trade index based on the BACI database including trade data of 75 countries. Accordingly, their paper constructed vertical trade map for the years of 2006 and 2007 [29]. Zhu et al. (2015) used a World Input-Output Database and presented global value networks (GVN), where the nodes are the individual industries in different countries and edges are the value-added contribution relationships. They also computed the global value trees (GVTs) by a bread-first search algorithm [30]. However, these studies did not fully adopt the latest outcomes of GVC accounting framework (e.g. KWW and WWZ), and therefore could not clearly visualize the features of global trade in details. Furthermore, as GVC accounting is deduced by the inter-countries input-output model under the general equilibrium assumption, the networks based on the accounting results are different from based on the international trade. For example, even there are no international trade between two countries, but there likely are value-added trade between them.

This paper makes two main contributions. Firstly we try to point out the differences between traditional international trade network and GVCs network, and summarizes the characters from the view of the whole network, inter-network, intra-network. Secondly we interpret the main networks features of the GVCs in equipment manufacturing industry by subdividing the entire GVC network into DVA (Domestic Value Added Absorbed Abroad) networks, RDV (Domestic Value Added First Exported then Returned Home) networks, FVA (Foreign Value Added) networks and PDC (Pure Double Counted Terms) networks. The additivity, correlation, topology and community evolution features of these networks are discussed in details.

## Methods

### Definition of GVC Networks

**Fig 1:**
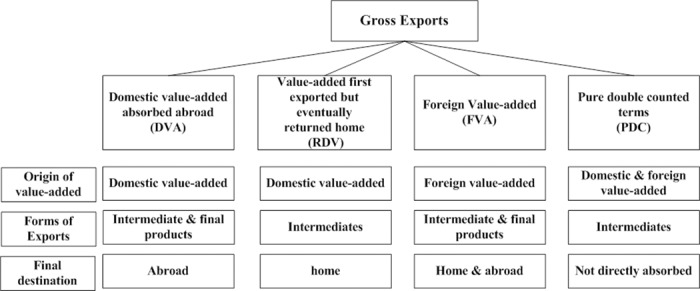
Framework of export decomposition.

According to the final absorbing country and absorbing approach, WWZ completely decomposed the bilateral trade flows at the sector level into 16 sections in added-value terms (Table A in S1 File). The 16 sections fall into 4 parts (Fig 1): Domestic Value Added Absorbed Abroad (DVA), Domestic Value Added First Exported then Returned Home (RDV), Foreign Value Added (FVA), and Pure Double Counted Terms (PDC).

There are several concepts we need to emphasize in advance. First, the export in gross terms at any level (country/sector level, overall country level, bilateral/sector level or overall bilateral level) can be completely separated into domestic value-added and foreign value-added. Second, the importing country is not necessarily the final absorbing country because of intermediate trade. This leads to multiple routines of value-addition in exports being absorbed (of the 16 components, 15 are associated with intermediate trade).

**Fig 2:**
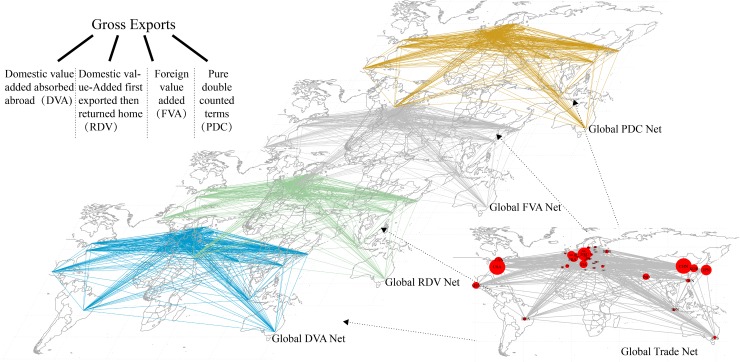
GVC networks and its decomposition.

Based on the complex network analysis tools, we can clearly know the flow of product from export country (starting node) to import country (destination node). The edges between nodes and the weight of edges respectively represent occurrence and magnitude of trade flow. However, according to the calculation method of WWZ, the gross export is denoted in added-value terms, and the relationship between export and import countries changes to a relationship through added-value flows. If we decompose gross exports into DVA, RDV, FVA, and PDC, the international trade networks can also be decomposed into DVA, RDV, FVA, and PDC sub-networks (Fig 2).

Based on the different concepts of the final absorbing country and the import country, and derived from the traditional trade networks under certain conditions, the GVC networks have the unique features:

1. Network features of' “general equilibrium”. The derivation in WWZ is based on the closed ICIO model that reflects the important view of supply-demand balance in the world market. The model also implies that the change of output (endogenous variable) is induced by the change of final demand (exogenous variable) through a chain transmission via trade in intermediate goods. This concept is vital for GVC framework and makes it possible to decompose trade volum in terms of value-added absorbed by various final demands. However, this feature may bring a challenge to the network analysis. The chain transmission yields a direct or indirect Input-Output relationship among countries and sectors. In other words, there must be edges among all countries if interactions are measured by value-added terms (illustrated in Fig 2). Intensive networks normally weaken the feasibility and reliability of complex network analysis. Therefore, we need to redefine the edges when using value-added terms [31].
2. Superposition and correlation of networks. Gross exports can be decomposed into DVA, RDV, FVA, and PDC. Accordingly, the international trade networks can be decomposed into four kinds of networks, which have features of network superposition (Fig 2). There may be interactive influences among these networks due to their different economic meanings and alternative or complementary relationships. Therefore, when evaluating the “superposition” of the network, the relative out-strength or in-strength of these four networks needs to be analysed, and the “correlation” of networks could reveal the interdependency among difference networks.
3. Heterogeneity of topology. Although complex networks are frequently used in traditional international trade studies, the topology in GVC networks differs from traditional trade networks in that the former emphasizes the relationships of value-added flows across countries. For instance, the DVA networks refer to the close relationships of domestic value added absorbed abroad between the export country and improt country, and the RVA networks refer to the close relationships of domestic value added first exported then returned home by re-imports between the export country and improt country. Also, from a dynamic view, the evolution of each GVC networks varies.

In summary, GVC networks can express international trade networks in the form of value-added flows under the GVC accounting framework. And GVC networks have the feature of “General Equilibrium”, superposition and correlation of networks, and heterogeneity of topology.

To construct GVC networks, we make the starting node *v*_*i*_ as the ith export country and the destination node *v*_*j*_ as the jth import country. The edge *a*_*i*,*j*_ is the flow of DVA, RDV, FVA or PDC from the ith export country to the jth import country denoted by the adjacency matrix A = [*a*_*i*,*j*_]. To ensure the sparsity of the matrix, *a*_*i*,*j*_ = 1 only if the DVA (or RDV, FVA, PDC) is greater than the median of the matrix A; otherwise *a*_*i*,*j*_ = 0. Because choosing different threshold values may lead to discrepancy of analysis results, we selected the mean, median, and upper four quartile as threshold values to make sensitivity analysis for more reliable results.

Table 1 shows that while we chose the mean, median, and upper quartile (the middle value between median and the highest value) as the threshold values to ensure the sparsity of the matrix, the order of countries placed in the top 10 of out-degrees had no obvious changes. Thus, it’s reasonable to believe that when we chose different threshold values, the core structure of GVC network of equipment manufacturing industry had no obvious changes. Moreover, we use the weight matrix W = [*w*_*i*,*j*_] to denote the magnitude of value-added flow from the ith export country to the jth import country. Finally, the V, A, and W together constitute the GVC networks denoted as G = (V, A, W).

**Table 1:** The top 10 of out-strength under different thresholds.

		1	2	3	4	5	6	7	8	9	10
DVA	Mean	CHN	DEU	USA	JPN	KOR	FRA	ITA	GBR	MEX	TWN
	Median	CHN	DEU	USA	JPN	KOR	FRA	ITA	GBR	MEX	TWN
	Q3	CHN	DEU	USA	JPN	KOR	FRA	ITA	GBR	MEX	TWN
RDV	Mean	USA	CHN	DEU	JPN	FRA	GRA	ITA	CAN	MEX	KOR
	Median	USA	CHN	DEU	JPN	FRA	GRA	ITA	CAN	KOR	MEX
	Q3	USA	CHN	DEU	JPN	FRA	GRA	ITA	CAN	KOR	MEX
FVA	Mean	CHN	DEU	TWN	KOR	MEX	USA	FRA	JPN	GBR	CZE
	Median	CHN	DEU	TWN	KOR	USA	MEX	FRA	JPN	GBR	ITA
	Q3	CHN	DEU	TWN	KOR	MEX	USA	FRA	JPN	GBR	CZE
PDC	Mean	CHN	DEU	TWN	KOR	MEX	FRA	JPN	CEZ	MEX	GBR
	Median	CHN	DEU	TWN	USA	KOR	FRA	JPN	CEZ	MEX	GBR
	Q3	CHN	DEU	TWN	KOR	USA	FRA	JPN	CEZ	MEX	GBR

Notes: Mean: the mean of matrix as threshold; Median: the median of matrix as threshold; Q3: the upper quartile of matrix as threshold.

### Indexes of GVC Network Analysis

According to the “General Equilibrium”, superposition and correlation of networks, and heterogeneity of topology, we selected the out-strength and in-strength to interpret the superposition of networks. We then selected the QAP (Quadratic Assignment Procedure) to interpret the interdependency of various networks as well as the reciprocity, assortativity, and community to interpret the heterogeneity feature of topology. The main indexes of the methods are as follows:

1. Out-strength. In GVC networks, the out-strength denotes the sum of value-added flows that a certain node sends to others. It measures weighted connectivity and gives us an idea of countries' value-added exports, i.e, shares in the world market, which reflects the ability to export [32]. We denote the out-strength as $S_{i}^{out}$, and it is calculated as:

$$S_{i}^{out}=\sum_{j}w_{i,j}$$ (1)

2. In-Strength. The in-strength denotes the sum of value-added flows that a certain node receives from other nodes. It is also measures weighted connectivity and gives us an idea of countries' value-added imports, which reflects the ability to receive. We denote the in-strength as $S_{i}^{in}$, and it is calculated as:

$$S_{i}^{in}=\sum_{j}w_{j,i}$$ (2)

3. Reciprocity. In GVC networks, there are three types of connection between two nodes: (1) non-connection, (2) non-reciprocal connection (only one node has edge to the other), and (3) reciprocal connection (the two nodes both have edges to the other). The reciprocity is denoted as the size of type (3) divided by the sum of type sizes (2)+(3). It is the tendency of countries to be economically interdependent and connected by two mutual links pointing in opposite directions. It is a particular type of correlation found in the international trade network that reflects the extent of reciprocity. With reference to Garlaschelli (2004) [33], the reciprocity index can be obtained by Eq (3), where $\bar{a}=\sum a_{i,j}/N(N-1)$.

$$Reciprocity=\frac{\sum_{i\neq j}\left(a_{i,j}-\bar{a})(a_{j,i}-\bar{a}\right)}{\sum_{i\neq j}{\left(a_{i,j}-\bar{a}\right)}^{2}}$$ (3)

4. Assortativity. According to Newman(2002), the assortativity coefficient measures the level of homophily of networks, and it is a scalar between -1 and 1. If the coefficient is high, then one node tends to link to other nodes with the same or similar strength (sum of in-strength and out-strength) and vice versa [34]. It may well happen that countries holding many links only trade with poorly-connected countries (we call such a network "disassortative"). Conversely, it may be the case that better connected countries also tend to trade with other well-connected countries (i.e. an "assortative" network) [35]. It is calculated by Eq (4), where H denotes the sum of weights of all edges in the network, *j*_*i*_ and *k*_*i*_, respectively, denote the strengths for the two nodes connected by link *i*.

$$Assortativity=\frac{H^{-1}\sum_{i}j_{i}k_{i}-{\left(H^{-1}\sum_{i}\frac{1}{2}(j_{i}+k_{i})\right)}^{2}}{H^{-1}\sum_{i}\frac{1}{2}(j_{i}^{2}+k_{i}^{2})-\left(H^{-1}\sum_{i}\frac{1}{2}(j_{i}+k_{i})\right)}$$ (4)

5. Community. Modularity is commonly used to evaluate the quality of the community division of networks. We adopt the algorithm developed by Blondel (1991) to calculate the modularity, which measures the density of links inside community compared to the links between communities [36]. It is a scalar between -1 and 1 and can be calculated by:

$$Q=\frac{1}{2m}\left[w_{i,j}-\frac{A_{i}A_{j}}{2m}\right]\delta (c_{i},c_{j})$$ (5)

where *A*_*i*_ = ∑_j_*w*_*i*,*j*_ is the sum of weights for edges attached to node i. If node i and node j are in the same community, *δ*(*c*_*i*_,*c*_*j*_) is 1; 0 otherwise. Where, *m* = ∑_*i*,*j*_*w*_*i*,*j*_/2.

To detect the communities, two processes are repeated iteratively. First, each node is considered to be a community. Thus, there are as many communities as the nodes. Then, according to Eq (6), we calculate the gain of modularity Δ*Q* for node i when it is placed into its neighboring community of j. Considering every neighboring community of node i, if the gain is negative then node i stays in its original community. If the gain is positive, then the i node joins the community with maximum Δ*Q*. This process is carried out repeatedly and sequentially for all nodes until no further improvement can be achieved, and the first process is then ended. Δ*Q* is calculated as:

$$\Delta Q=\left[\frac{\sum C_{in}+A_{i,in}}{2m}-{\left(\frac{\sum tot+A_{i}}{2m}\right)}^{2}\right]-\left[\frac{\sum in}{2m}-{\left(\frac{\sum tot}{2m}\right)}^{2}-{\left(\frac{A_{i}}{2m}\right)}^{2}\right]$$ (6)

where ∑*C*_*in*_ is the sum of weights of all edges inside the community C, ∑*tot* is the sum of all edges connected to the nodes in the community C, *A*_*i*_ is the sum of weights of edges connected to the nodes i, *A*_*i*,*in*_ is the sum of weights of edges from node i to all nodes in community C, and m is the sum of weights of all edges in the network.

The second process of the algorithm is to construct a new network where the nodes belong to the communities detected in the first process. In the new network, the weights of edges between the new nodes are calculated by the sum of the weights of edges between the corresponding two communities. The edges between the nodes of the same community are seen as a self-loop in the new network. Once the second process is completed, the first process is reapplied to the obtained network. The two processes are iterated until there are no more changes [37].

It should be noted that, since "assortativity" and "community" are based on an undirected version of the network, we transform the directed network into an undirected one by adopting the mean value of both directions.

## Results and Analysis

### Basic Topology of GVC Networks

**Fig 3:**
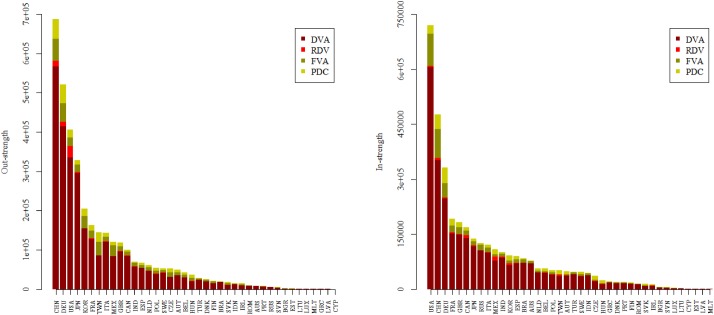
The out-strength and in-strength of GVC networks (2011).

Using the WIOD and selecting the sectors 13–16 (equipment manufacturing sector) [38], we first calculated out-strength and in-strength of DVA, RDV, FVA and PDC sub-networks for all countries. Fig 3 shows these results for 2011. In general, both out-strength and in-strength for a specific country in the corresponding networks depend on the size of the country. However, for each sub-network, there is a large variation across countries at the absolute level. In terms of DVA, the out-strengths of China, Germany, United States and Japan are significantly higher than that of other countries, indicating that the four countries act as the main senders of value-added in the DVA networks. On the other hand, the in-strength of the United States, China and Germany are higher than other economies, which implies that these three countries absorb more value-add from direct trade partners. The RDV out-strength of United States is obviously larger than that of other countries because US engages in product design and core parts production in the equipment manufacturing related GVCs. A considerable part of value-add is embodied in its intermediate goods and services that are exported to other countries for further processing and finally re-imported and consumed in the domestic market.

The RDV in-strength of China, Canada and Mexico are relatively larger than other countries. The reason is that all the three countries are the main trade partners of the United States through fragmentation production networks. That’s to say, the large out-strength of RDV for the United States is achieved mainly by the way of intermediate trade with China, Canada and Mexico. For the out-strength of FVA, China, Germany, Korea, Chinese Taipei and Mexico show a high absolute presence. This is because these economies import a massive amount of intermediates to produce exports in GVCs. For the in-strength of FVA, the United States, China and Germany have the largest figures. For both the out-strength and in-strength in PDC, China and Germany are outstanding. This reflects the fact that both China and Germany are the world’s manufacturing centers. To be more specific, China and Germany are the cores of East Asia and Europe’s production networks respectively, and a large number of intermediate trade via these two countries drives up the proportion of double-counting in international trade.

**Fig 4:**
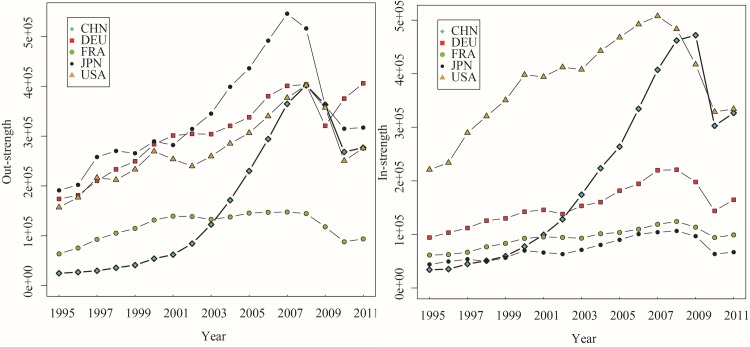
The out-strength and in-strength of DVA networks (1995–2011).

**Fig 5:**
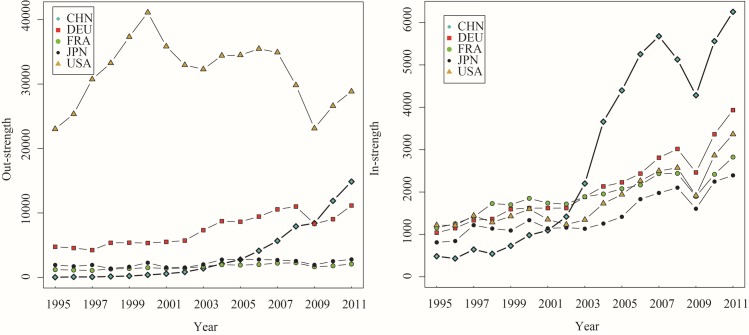
The out-strength and in-strength of RDV networks (1995–2011).

**Fig 6:**
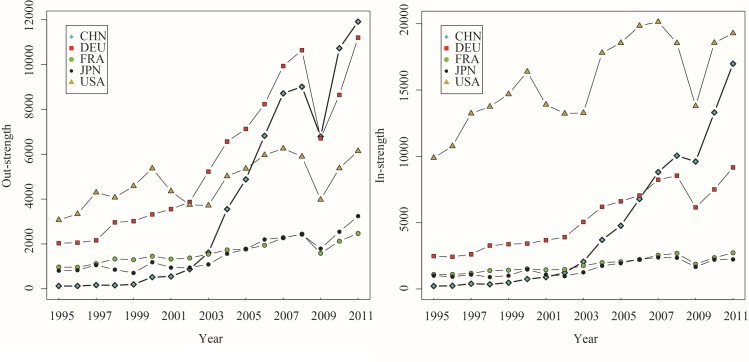
The out-strength and in-strength of FVA networks (1995–2011).

**Fig 7:**
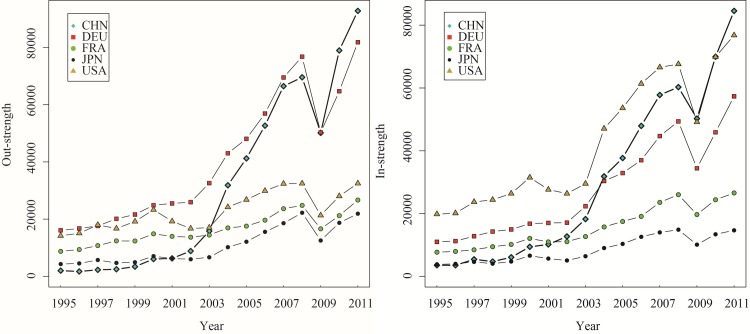
The out-strength and in-strength of PDC networks (1995–2011). The WIOD data is based on the current dollar. To avoid the effect of inflation on dynamic analysis, we deflated total output and value added to get the 16 items of trade flow subdivision of WWZ after leveling, according to the WIOD price table and social accounts deflator (relative to the 1995 total output price index and the value added price index). This assumes unchanged structure of input-output and considers changed exchange rates. We obtained out-strength and in-strength of networks of DVA、RDV、FVA、and PDC from 1995 to 2011 (Figs 4–7).

Next, we analyze the evolution of DVA, RDV, FVA, and PDC’s out-strength and in-strength for China, Germany, France, Japan, and the United States from 1995 to 2011. As shown in Figs 4–7, these countries’ in-strengths and out-strengths continued rising except for a slight decrease in 2008 due to the financial crisis. Then, the GVC networks became increasingly dense. Interestingly, different from the overall trend, the RDV out-strength of the United States rose first, then dropped, and rose up again. One possible explanation is that, before 2000, United States’ manufacturing sector had obvious technological advantages, focusing its activities on product design and production of core parts at the upstream of the value chain. A considerable part of value-added in exports returned and was finally consumed domestically. After 2000, the United States once viewed the manufacturing as a “sunset industry”, and moved the production capacity, assembly and even a part of R&D abroad. After the financial crisis, the United States adopted the “re-industrialization” strategy by being the supplier of high-end parts in manufacturing sector.

The DVA out-strength of China grew up rapidly, caught up with that of Germany and jumped to the first place in 2008. At the same time, the FVA, PDC out-strengths of China also surpassed those of other countries, which reflects the fact that China’s exports included many foreign value-added and double-counting parts. Since 2003, the RDV in-strength of China remained significantly higher than those of other countries indicating that China’s imports included many intermediate products and these intermediate products were exported to other countries again after domestic processing. However, there is still a big gap between the RDV out-strengths of China and United States, and only a little value-added of the intermediate exports return China. In comparison, the United States, as the world’s largest market, has greater DVA, FVA in-strengths than other countries, and is an important destination for final goods in GVC networks.

The DVA、RDV、FVA and PDC have significant correlation, and the correlation coefficient tends to rise, which illustrates that different forms of value added trade networks may be increasingly relevant (Table 2). The correlation coefficients among DVA、FVA、PDC networks are all above 0.7, but the correlation coefficients between RDV network and other networks (particularly DVA and RDV) are significantly lower, which indicates the status of a country in RDV network is not necessarily the same as the status of a country in DVA、RDV and PDC network. For example, the countries at the high-end of the value chain have large DVA, FVA, and PDC and also have large RDV; however, the countries at the low-end of the value chain have large DVA, FVA, PDC, but may have small RDV.

**Table 2:** The QAP correlation coefficient matrix of DVA, RDV, FVA, and PDC networks in 1995, 2000, 2005, and 2011.

**1995**	**DVA**	**RDV**	**FVA**	**PDC**	**2000**	**DVA**	**RDV**	**FVA**	**PDC**
DVA	—	—	—	—	DVA	—	—	—	—
RDV	0.389	—	—	—	RDV	0.412	—	—	—
FVA	0.720	0.363	—	—	FVA	0.770	0.374	—	—
PDC	0.716	0.665	0.884	—	PDC	0.734	0.672	0.870	—
**2005**	**DVA**	**RDV**	**FVA**	**PDC**	**2011**	**DVA**	**RDV**	**FVA**	**PDC**
DVA	—	—	—	—	DVA	—	—	—	—
RDV	0.402	—	—	—	RDV	0.483	—	—	—
FVA	0.761	0.278	—	—	FVA	0.783	0.336	—	—
PDC	0.652	0.441	0.862	—	PDC	0.667	0.469	0.884	—

Notes: All correlation coefficients are significant at 1% confidence.

**Fig 8:**
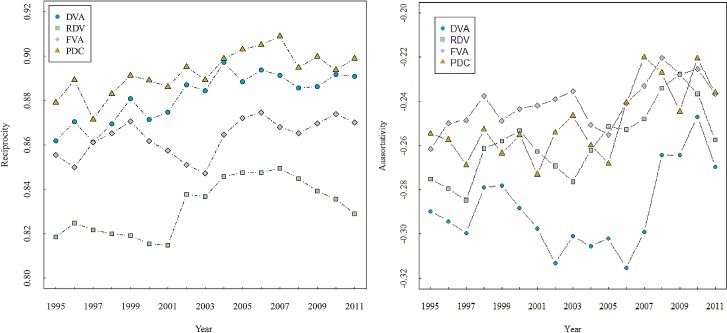
The reciprocity and assortativity of DVA, RDV, FVA, and PDC networks (1995–2011).

According to Eqs (3) and (4), we calculate the reciprocity and assortativity of DVA, RDV, FVA, and PDC networks from 1995 to 2011. Fig 8, shows that the reciprocal edges accounted for more than 80% of the total edges. This indicates the reciprocity of networks. The reciprocity of DVA, RDV, FVA, and PDC increased over time, but there was a significant decline in 2007—especially for the RDV network probably due to the financial crisis. The assortativities of DVA, RDV, FVA, and PDC networks are all below zero, showing that the countries with large strengths tend to attach to countries with small strengths. Due to the geographical proximity and cultural similarity, the regional small countries tend to trade with the hub countries in this region, which contributes to the formation of regional value chains with powerful countries as cores such as the EU, NAFTA and APEC, etc. However, with the rapid development of the globalization, any regional value chains cannot disconnect with other regions. This results in the situation whereby the regional core countries serve as hubs, and bridges when connecting to other regions.

### Communities Evolution

**Fig 9:**
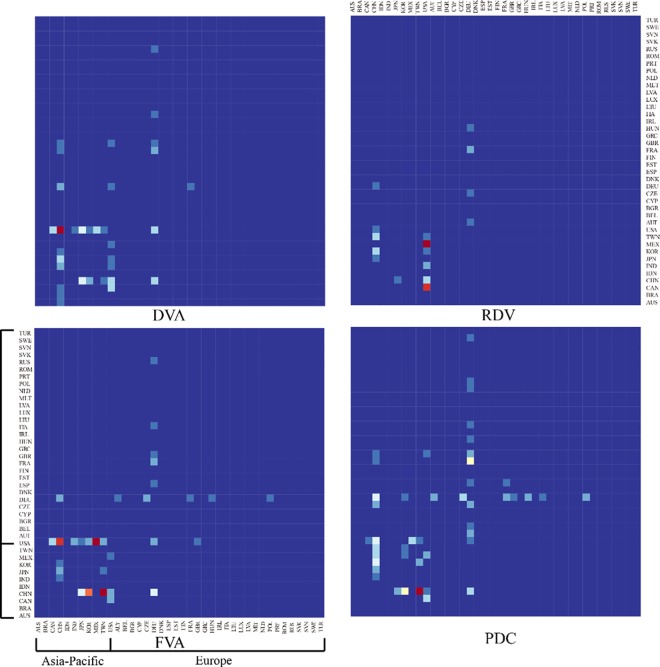
The connections of DVA, RDV, FVA, and PDC networks (2011). Notes: Colors scale (changing from blue to red) represents growing value-added of DVA, RDV, FVA, and PDC. The horizontal axis represents sender of value-added flows, and the vertical axis represents receiver of value-added flows.

Fig 9 shows the connections among DVA, RDV, FVA, and PDC networks in 2011. It is not difficult to find the grids with colors close to red are mainly among the Asia-Pacific countries (including China, Japan, Korea, Chinese Taipei, India, Indonesia, the United States, Canada, Mexico, Brazil, and Australia). This indicates that value-added flows among Asia-Pacific countries are denser than those among other countries. There may be communities in the equipment manufacturing GVC networks.

**Fig 10:**
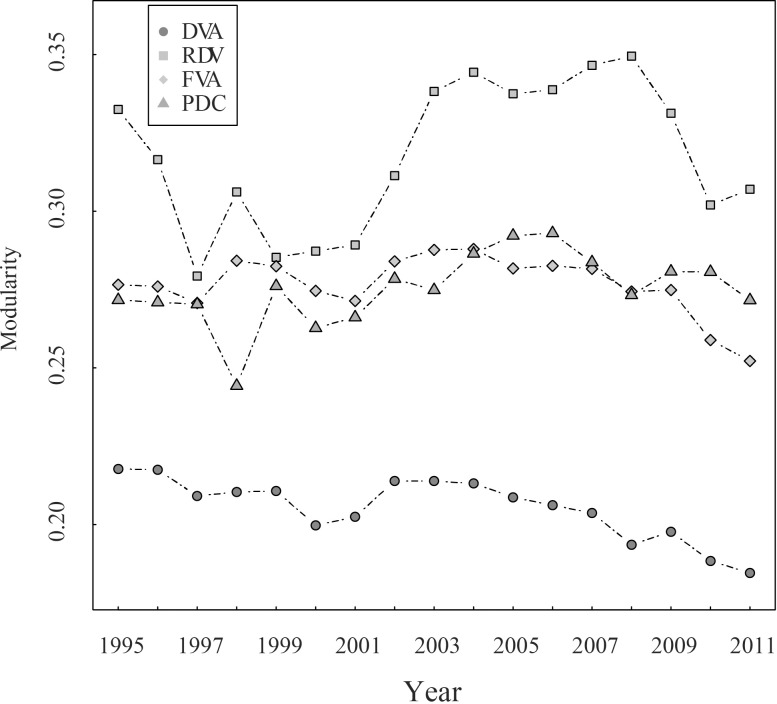
The modularity of DVA, RDV, FVA, and PDC networks (2011).

To verify our hypothesis, we used Eqs (5) and (6) to calculate the modularity of DVA, RDV, FVA, and PDC networks and then analyzed the communities in these networks. Although the divisions of communities are different in some years (caused by certain small countries drifting across communities), the communities are overall stable in each network. The memberships of the communities remained essentially unchanged. China, Japan, South Korea, Chinese Taipei, India, Indonesia, the United States, Canada, Mexico, Brazil, and Australia constitute the first community—the Asia-Pacific community. Germany, France, Britain, Italy and other European countries constitute the European community. The modularity of the RDV network is significantly higher than other networks (Fig 10). The reason behind is that RDV reflects the special trading mode that one country exports intermediate products to other country, and then the added value returned and is finally consumed domestically, which leads to a much complex trade connection among countries in the same community.

The modularity of DVA network is the lowest because the domestic value-added embodied in final good trade is the majority in DVA, which reflects a very direct connection between trade partners. Therefore, the communities in the DVA network is not so obvious. Generally, the modularities of DVA, RDV, FVA, and PDC networks show a downward trend indicating that the cliques of the GVC networks are getting loose.

The GVC networks are too dense for us to visualize some important topology features (such as the community, hierarchy, core–periphery). Zhou et al. (2016) classified the network by the import or export ranking of each country and retained only the top-ranking importers or exporters to construct the network for preserving only the basic information of the network. At the same time, they simplified the network to analyze the characteristics of the network topology [31]. Specifically, we try to form a very significant tree structure by defining the “top 1” import network, which is the network only retaining the top 1-ranking import relation of a country. Then we can analyze the evolution of the GVC network topology through the visualization of the DVA, RDV, FVA, and PDC’s top 1 network.

**Fig 11:**
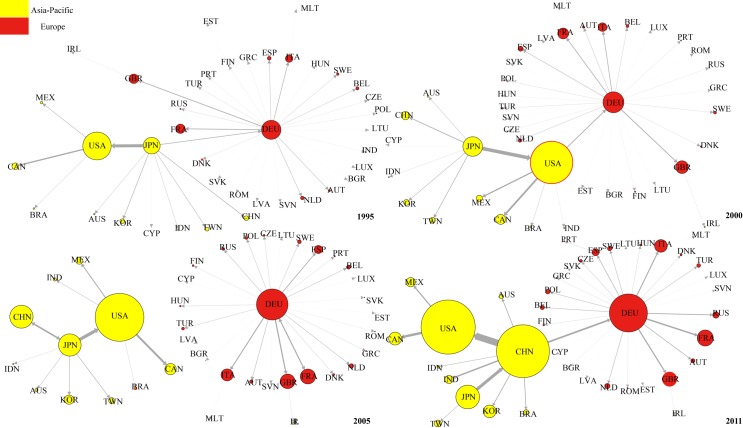
The evolution of DVA network (top 1). Notes: Nodal size is proportional to the out-strength. Edge width is proportional to strength of value-added flow. Given the large value-added of DVA and FVA, to facilitate drawing, the nodes in the DVA, FVA networks represent 20 times and 2 times the strength of the nodes of the same size in RDV or PDC network.

Fig 11 shows the evolution of top 1 DVA network from 1995 to 2011. We found that in 1995, Japan was the hub of the entire DVA network connecting the Asia-Pacific community (with the United States as the core) and European community (with Germany as the core). Japan was also the core in East Asia coinciding with the so-called “flying geese pattern” [39]. From 2000–2005, with the decline of Japanese economy, the “flying geese pattern” gradually disintegrated, and the United States became the new hub of DVA network while Japan remained the core in East Asia.

In 2011, having replaced the United States as the hub of DVA network and being a new core in East Asia, China developed into a “world factory” and has even become one of the cores of the global DVA network. The evolution process of the DVA network is very similar to the results of Zhu, et al. (2014). That is, China gradually overtook Japan to become the core of the network of Asia-Pacific Trade [40].

**Fig 12:**
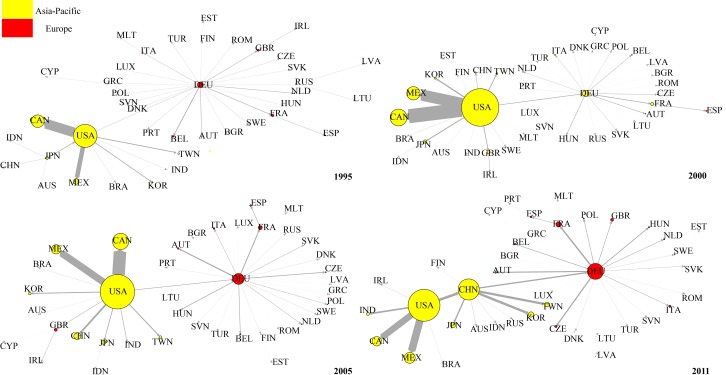
The evolution of RDV network (top 1).

Fig 12 shows the evolution of the top 1 RDV network. In contrast to the DVA network, from 1995 to 2005, the United States was remained the core of the Asia-Pacific community and connected the Asia-Pacific community to the European community (with Germany as the core). This is because considerable intermediate products made in the US at the high-end of the value chain are exported to other countries, assembled into final products, and then returned the domestic market. This reflects the United States’ dominance in the value chain. China gradually improved its position in the RDV network. In 1995, China was at the peripheral position of the RDV network and gradually transferred to the middle position from 2000 to 2005, and became the hub connecting the Asia-Pacific community and European community in 2011. However, please note that the arrow pointing to China from the United States suggesting that the United States is China’s largest RDV exporter. On the other hand, China’s RDV out-strength is still significantly less than that of the United States, indicating a great gap between China and the United States in terms of position in RDV network.

**Fig 13:**
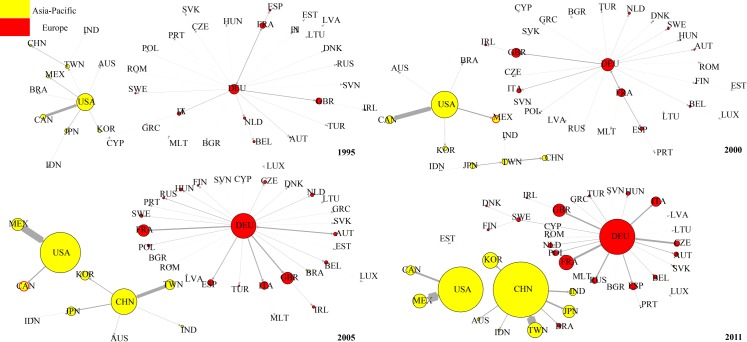
The evolution of FVA network (top 1).

Fig 13 shows the evolution of top 1 network for FVA. Compared with the DVA and RDV networks, the FVA network is more dispersed, and the European community (Germany always functions as the core) has no connection with the Asia-Pacific community. In 1995, the United States was the core of the Asia-Pacific community. In 2000, China, Japan, Chinese Taipei, India, and Indonesia presented a “chain” connection, while the United States continued to be the core of other Asia-Pacific countries. In 2005 and 2011, the United States only maintained connections with Canada and Mexico. China became the new core of the Asia-Pacific community.

This evolution shows that the manufacturing capacity of developing countries in East Asia were initially weak, and part of their production processes at the low end of value chain still needed to be done in the developed countries. Thus there were connections among almost all countries in the Asia-Pacific community in 1995. As East Asia’s developing countries were increasingly improving their industrialization, China, India and other East Asian developing countries established a regional complementary relationship of production chain with Japan, Chinese Taipei and other emerging countries in the Asia-Pacific community. After 2005, as China developed into a “World Factory”, China moved up to the core of East Asia’s FVA network, and the United States remained connected only to its neighboring countries such as Canada and Mexico.

**Fig 14:**
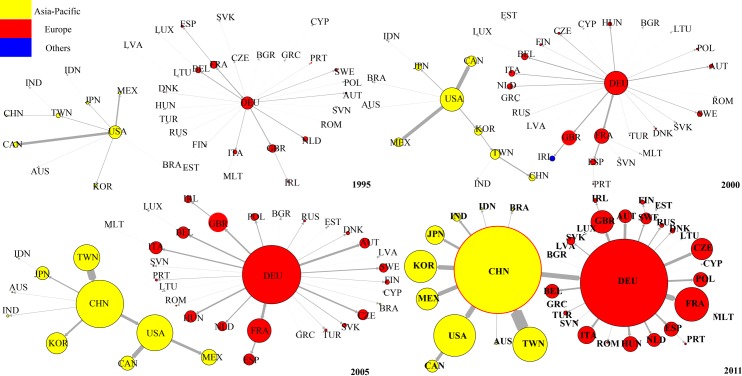
The evolution of PDC network (top 1).

Fig 14 shows the evolution of the top 1 PDC networks from 1995 to 2011. One important feature of the PDC networks is the obvious “chain” structure among countries. (The chain structure indicates that the network is hierarchical while the star structure indicates that the network is flat according to Shi (2014) [41]). This is because PDC mainly appears in the intermediate goods trade where the chain structure is very significant. Moreover, Germany has always been the core of the European community countries. The United States was the core of the Asia-Pacific community, and China was on the periphery. In 2005 China became the core in East Asia connected with the United States, but there was still no connection between the Asia-Pacific community and the European community. In 2011, China became the core of the Asia-Pacific community and became Germany’s largest PDC exporter. Thus a bridge was established between the Asia-Pacific community and the European community. Another important feature of the PDC network is that the nodes expand rapidly indicating that the development of the intermediate goods trade made the portion of double counting in trade grow rapidly under global production fragmentation.

Over all, similar to Cerina et al. (2015) [8], the GVC networks in the European community was led by Germany. To something different, other countries in Asia-Pacific region made up the Asia-Pacific community and Japan, the United States and China became the core of the Asia Pacific community successively through the sample interval.

To reflect the relationship between countries and roles of different communities, we calculated the value-added flowing among and inside the communities, and measured the countries’ out-degrees of the Top 1 network. As shown in Table 3, the ratio of DVA, RDV, and FVA flowing to the inside of the Asia-Pacific communities fell from 70.24%, 85.16%, and 75.73% in 1995 down to 69.33%, 74.75% and 72.83% in 2011. The out-degree of the top 1 network were from 0 up to 8, 9, and 10 at the same time. In other words, China was the largest DVA, RDV and FVA exporter of 8, 9 and 10 countries in the DVA, RDV and FVA networks in 2011. This shows that the proportion of added value from China to the Asia-Pacific region declined slightly, but China’s control of the key value flow significantly increased. All out-degrees (in the top 1 network) of the US and Japan declined, but the added value flowing to the Asia-Pacific Community still maintained a high proportion of total added value (particularly RDV network). This means that the impact of the US and Japan on the equipment manufacturing industry GVC network is waning, but the Asia-Pacific region is still an important area for their equipment manufacturing industry’s production and trade.

**Table 3:** The ratio of value-added flow to the inside of the communities and the top 1 network out-degrees of major countries.

Community		Asia-Pacific				Europe		
country		CHN	USA	JPN	KOR	DEU	FRA	GBR
DVA	1995	70.24%(0)	64.91%(4)	73.89%(8)	71.91%(0)	66.67%(25)	73.40%(0)	62.12%(1)
	2000	72.14%(0)	64.16%(6)	74.94%(7)	73.26%(0)	62.55%(25)	70.06%(0)	55.16%(1)
	2005	72.22%(1)	66.33%(4)	73.61%(6)	65.99%(0)	63.24%(26)	71.10%(1)	58.50%(1)
	2011	69.33%(8)	71.18%(2)	75.00%(2)	71.10%(0)	57.98%(25)	65.99%(0)	66.67%(1)
RDV	1995	80.16%(0)	93.82%(8)	86.28%(4)	87.64%(0)	95.11%(21)	96.55%(1)	92.42%(1)
	2000	77.78%(0)	92.01%(14)	86.88%(1)	86.11%(0)	94.54%(20)	95.78%(1)	90.35%(1)
	2005	79.34%(0)	92.96%(11)	90.75%(1)	88.56%(0)	93.06%(22)	95.15%(2)	91.08%(2)
	2011	74.75%(9)	92.29%(6)	89.70%(0)	88.22%(0)	90.97%(19)	94.51%(2)	87.55%(0)
FVA	1995	75.73%(0)	69.97%(7)	81.69%(1)	83.55%(0)	69.60%(18)	72.45%(2)	59.84%(1)
	2000	73.68%(0)	69.88%(5)	81.69%(2)	83.97%(0)	66.56%(21)	71.10%(3)	51.69%(1)
	2005	74.94%(5)	71.67%(2)	82.39%(1)	78.95%(0)	64.67%(26)	69.97%(0)	57.27%(1)
	2011	72.83%(7)	76.91%(2)	84.03%(0)	84.54%(0)	57.45%(22)	67.00%(0)	52.38%(1)
PDC	1995	60.16%(0)	73.33%(7)	69.97%(1)	71.75%(0)	84.23%(20)	85.53%(2)	80.54%(1)
	2000	59.02%(0)	73.19%(6)	70.93%(1)	72.38%(1)	81.85%(21)	83.82%(4)	76.02%(1)
	2005	61.09%(6)	75.19%(2)	75.55%(1)	73.47%(0)	83.19%(25)	83.11%(2)	77.27%(1)
	2011	60.32%(10)	71.10%(1)	73.40%(0)	73.68%(0)	78.63%(23)	81.69%(1)	75.85%(1)

Notes: The figures outside the brackets are the ratio of value-added flow to the inside of the communities and those in the brackets are the top 1 network out-degrees of main countries.

The proportion of output of Germany, France and Britain to the European Community’s added value reduced. This was not because much of European added value decreased, but mainly because the attractiveness of added value in Asia-Pacific communities grew rapidly. For example, in the sample interval, the RDV output of Germany to France increased 1.90 times and the RDV output of Germany to China increased 28.06 times. As the largest added value exporter country among many European countries, Germany’s out-degree was much higher than that of other countries. In contrast, the impact of the French and the UK was limited in the local area. For example, the United Kingdom was only Ireland's largest exporter country of value added, and France was Spain's largest RDV exporter country.

**Fig 15:**
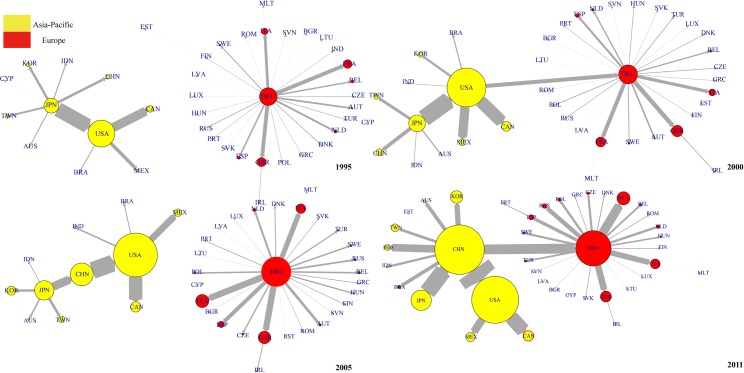
The evolution of traditional trade network.

We sum the 16 items of trade flow subdivision of WWZ to get traditional trade flows (WWZ thoroughly subdivides bilateral trade flows of department level into 16 value added groups, so by summing the 16 items we can get traditional trade flows). Accordingly, we construct the traditional trade network of equipment manufacturing industry and investigate its evolution. The evolution of Top 1 network of traditional trade network from 1995 to 2011 is shown in Fig 15. Generally speaking, the topological structure of traditional trade Top 1 network is a little similar to DVA Top 1 network’s, perhaps due to the fact that the DVA occupies a larger proportion in traditional trade flows. However, the structures of RDV, FVA,and PDC top 1 network are different from traditional trade network. For example, in 2005, China was in the peripheral position of RDV Top 1 network, but was the hub in traditional trade network that connected Japan and the USA. In addition, the “chain” structure among countries in PDC and FVA networks was not reflected in the traditional trade network. Therefore it is meaningful for us to observe interesting characteristics concealed in the traditional trade network through a GVC network.

## Conclusions

Based on the decomposition of bilateral gross exports in value-added terms proposed by KWW (2014) and WWZ (2013), this paper visualized the 1995–2011 equipment manufacturing-related GVC networks in terms of four participation patterns of GVCs (DVA, RDV, FVA and PDC). It combines complex network analysis techniques, and analyzes the basic topology and community evolution of the corresponding networks. We conclude: (1) Under the global GVC accounting framework, the GVC networks represent the network of embodied value-added flows across countries, which has the “general equilibrium”, superposition, and correlation features of networks as well as heterogeneity of topology. (2) The DVA out-strengths of China, Germany, United States and Japan are significantly higher than those of other countries indicating that the four countries are senders of value-added flows in the DVA network. The FVA, PDC out-strengths of China are larger than those of the United States, while there is a wide gap between the out-strength of China and the United States. This shows that the United States still has an obvious advantage in Value Chain by providing high complex intermediate goods. (3)The DVA, RDV, FVA and PDC have significant correlation, and correlation coefficient tends to rise, which illustrates that different forms of value added trade networks may be increasingly relevant. (4) The DVA, RDV, FVA, and PDC networks express the feature of reciprocity. However, the assortativities of DVA, RDV, FVA, and PDC networks are all below zero, which means that countries with large strength tend to attach to countries with small strength. (5) The communities are overall stable in the DVA, RDV, FVA, and PDC networks, and memberships of communities are essentially unchanged. China, Japan, South Korea, Chinese Taipei, India, Indonesia, the United States, Canada, Mexico, Brazil, Australia constitute the Asia-Pacific community. Germany, France, Britain, Italy and other European countries constitute the European community. This somewhat implies that the geographic distance still matters in GVCs. More dynamic changes happen inside regional value chains. (6) We can identify different evolutionary characteristics in the so-called top 1 network of DVA, RDV, FVA, and PDC. For example, the FVA network is more discrete, and PDC network presents obvious complex “chain” structure.

## Supporting Information

S1 File Appendix. (DOC)
