# Supplementary material for: Complex Network Analysis for Characterizing Global Value Chains in Equipment Manufacturing
Source: PLoS One. 2017 Jan 12;12(1):e0169549. doi: 10.1371/journal.pone.0169549 (PMC5231281; doi:10.1371/journal.pone.0169549)
Supplement: S1 File — (DOC) [file pone.0169549.s001.doc]

# S1 Appendix：

The full decomposition of Country s' export to Country r in WWZ(2013)

**(A1)**

Suppose there are Country r, s, t. The exports from Country s to Country r $E^{sr}$ can be decomposed into two parts: final goods exports $\text{Y}^{\text{sr}}$ and intermediate goods exports $Z^{\text{sr}}$, which can be expressed as the direct input output coefficient $A^{sr}$ multiply by the output of Country r using Leontief model. WWZ(2013) fully decomposes $E^{sr}$into 16 item as shown in formulas A1, where V denotes direct value-added coefficient vector, B denotes total IO coefficient, Y denotes final demand, T denotes matrix transpose operation, # denotes element-wise matrix multiplication operation, L denotes local Leontief inverse, $E^{r*}$denotes the total exports of Country r. For each term, the part before # means where is the value added from, the part after # means where is the value added absorbed. The definition of the 16 terms in equation A1 is described in Table A in S1 File.

Summing the T1, T2, T3,T4,T5 yields domestic value-added absorbed abroad (DVA), summing the T6,T7 and T8 yields domestic value-added returns home (RDV), summing the T11,T12, T14 and T15 yields foreign value-added (FVA), summing the T9,T10, T13 and T16 yields pure double counted Terms (PDC).

**Table A** Definition of the 16 Terms in Equation

| Label | Description |
| --- | --- |
| T1 | DVA exports in final goods exports |
| T2 | DVA in intermediate exports to the direct importer and is absorbed there |
| T3 | DVA in intermediate exports used by the direct importer to produce intermediate exports for production of third countries’ domestic used final goods |
| T4 | DVA in Intermediate exports used by the direct importer producing final exports to third countries |
| T5 | DVA in Intermediate exports used by the direct importer producing intermediate exports to third countries |
| T6 | Returned DVA in final goods imports -from the direct importer |
| T7 | Returned DVA in final goods imports -via third countries |
| T8 | Returned DVA in intermediate imports |
| T9 | Double counted DVA used to produce final goods exports |
| T10 | Double counted DVA used to produce intermediate exports |
| T11 | Direct importer’s VA in source country’s final goods exports |
| T12 | Direct importer’s VA in source country’s intermediate goods exports |
| T13 | Direct importer’s VA double counted in exports production |
| T14 | Third countries’ VA in final goods exports |
| T15 | Third countries’ countries’ VA in intermediate goods exports |
| T16 | Third countries’ VA double counted in exports production |
